# Grade 4 Pneumonitis in a Patient Treated with a Combination of Gemcitabine and Docetaxel for Recurrent Leiomyosarcoma of the Uterus

**DOI:** 10.1155/2020/4629452

**Published:** 2020-02-07

**Authors:** Connor Wang, Stephen Rose, Lori Mankowski Gettle, Ryan Spencer

**Affiliations:** ^1^Department of Obstetrics and Gynecology, University of Wisconsin School of Medicine and Public Health, Madison, WI 53792, USA; ^2^Division of Gynecologic Oncology, University of Wisconsin School of Medicine and Public Health, Madison, WI 53792, USA; ^3^Department of Radiology, University of Wisconsin School of Medicine and Public Health, WI 53792, USA

## Abstract

Gemcitabine and docetaxel combination chemotherapy is the standard of care for patients with unresectable recurrent or metastatic leiomyosarcoma of the uterus. Although they are generally well-tolerated agents, they can also cause severe and life-threatening pulmonary toxicities. Here, we describe a case of grade 4 pneumonitis due to gemcitabine and docetaxel in a 74-year-old woman with recurrent, metastatic uterine leiomyosarcoma. Despite early recognition of chemotherapy-induced lung injury and early administration of corticosteroid, she developed noncardiogenic pulmonary edema, diffuse alveolar hemorrhage, and acute respiratory distress syndrome. She required multiple intubations and a tracheostomy. Physicians should not only be aware of gemcitabine and docetaxel's potential to cause life-threatening pulmonary injuries but also recognize the variability in clinical presentations and treatment responses, the radiographic findings of these lung toxicities, and the need for early corticosteroid therapy in these cases.

## 1. Introduction

Uterine leiomyosarcoma (uLMS) is the most common uterine sarcoma. It has an annual incidence of approximately 0.8 per 100,000 women with over 60% diagnosed at International Federation of Gynecology and Obstetrics (FIGO) stage I [1]. Although the majority are limited to the uterus on presentation, these tumors are highly aggressive and have a high recurrence rate. Initial treatment for early-stage disease is total hysterectomy (TH) with or without bilateral salpingo-oophorectomy (BSO) and lymphadenectomy depending on patient factors and the clinical scenario. For stage I patients, it is reasonable to consider either observation or adjuvant chemotherapy. Gynecologic Oncology Group- (GOG-) 0277 attempted to investigate the role of adjuvant chemotherapy for uLMS; however, this trial did not complete its targeted accrual, precluding comparison of survival outcomes in completely resected uLMS [2]. Additional prospective and retrospective data have shown observation with imaging to be equivalent to adjuvant chemotherapy [3].

In the recurrent/metastatic settings, multiple studies have demonstrated the efficacy and tolerability of gemcitabine/docetaxel (G/D) for uLMS. Notably, GOG-87L [4] and GOG-131G [5] demonstrated G/D as an active regimen for chemotherapy-naïve and for second-line treatment of advanced, unresectable uLMS, respectively. Further, GOG-250 [6] investigated the addition of bevacizumab in the treatment of chemotherapy-naïve, metastatic uLMS. This study closed for futility after demonstrating that bevacizumab did not improve outcomes. G/D remains the standard of care in this setting.

Gemcitabine (a pyrimidine analog) and docetaxel (a taxane antineoplastic agent) are used in a variety of solid tumors. Myelosuppression is the most common dose-limiting side effect for both agents [4, 5, 7]. Up to 25% of patients receiving gemcitabine may report adverse pulmonary symptoms, but these are generally grade I-II pulmonary toxicities that manifest as dyspnea but do not limit self-care [7]. Severe adverse pulmonary events, defined as ≥grade 3 by the National Cancer Institute for Common Terminology Criteria for Adverse Events (CTCAE), have been described in case reports or studies with both gemcitabine and docetaxel independently, as well as in combination. Here, we report a case of grade 4 pneumonitis after G/D combination therapy in a patient with recurrent uLMS. To our knowledge, this is the first reported case of such a severe pulmonary toxicity in a patient receiving G/D for recurrent uLMS.

## 2. Case Presentation

This case is of a 74-year-old Caucasian woman who was initially diagnosed with stage IB uLMS in May 2018 after she underwent an exploratory laparotomy, TH, and BSO. Intraoperative frozen pathology was notable for spindle cell neoplasm—unable to further characterize. The final pathology revealed uLMS (31 × 29 × 16 cm in size) confined to the uterus. Her past medical history included hypertension and hypothyroidism without significant cardiopulmonary history. She never received radiation to her thorax and never smoked. She opted for observation instead of adjuvant therapy following her surgery. Twelve months later, in May of 2019, a surveillance computed tomographic (CT) scan showed mesenteric and peritoneal masses without thoracic involvement. Her subsequent percutaneous biopsy demonstrated recurrent uLMS. She was started on intravenous (IV) gemcitabine 900 mg/m^2^ on cycle days 1 and 8 and IV docetaxel 100 mg/m^2^ on cycle day 8 every 21 days. Her chemotherapy course was complicated by neutropenia, requiring a dose reduction in docetaxel to 75 mg/m^2^ starting on cycle 3 day 8.

On day 19 of cycle 4 of G/D (eighty-two days after chemotherapy initiation), she presented to the emergency department (ED) with 1 week of cough and dyspnea at rest that interfered with her activities of daily living. Her review of systems was otherwise unremarkable. On physical exam, she was afebrile and had a pulse of 81 beats per minute (bpm), blood pressure of 95/56 mmHg, respiratory rate of 22 breaths per minute, and oxygen saturation (SaO_2_) of 88%. During her time in the ED, she had an escalating oxygen (O_2_) requirement necessitating up to 6 L/min via a nasal cannula. Her cardiac exam was normal. Her pulmonary exam demonstrated diminished breath sounds and crackles throughout her mid and lower lungs bilaterally. Her abdominal exam was normal. Her extremities showed trace nonpitting bilateral lower extremity edema.

**Figure 1:**
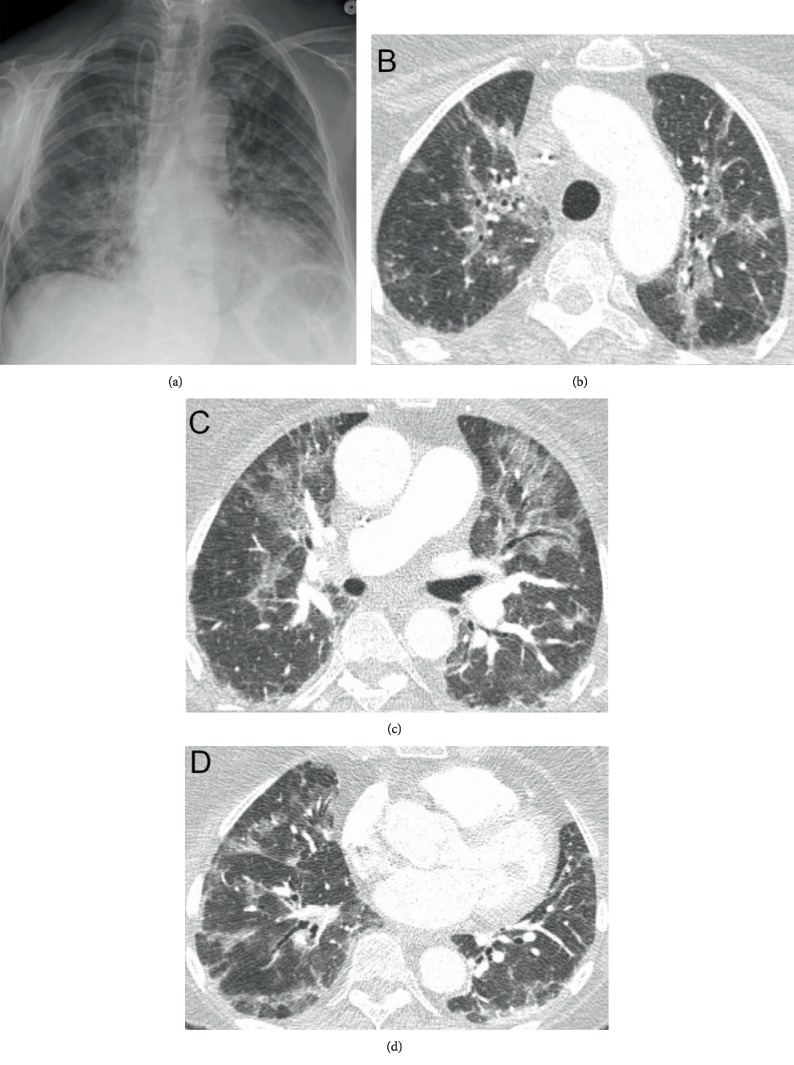
Imaging on presentation to ED. (a) CXR with multifocal airspace opacities. (b–d) CT scan of the lungs at upper (b), mid (c), and base (d) illustrating bilateral peribronchial consolidation and ground glass opacities.

Laboratory studies were significant for leukocytosis (18.2 K/*μ*L with 730 cells/*μ*L of immature granulocytes and an absolute neutrophil count of 13,650 cells/*μ*L). Her white blood cell count 4 days prior was 4.8 K/*μ*L. Her creatinine was 0.87 mg/dL (previously 0.69 mg/dL), and she was hyponatremic (129 mmol/L) and hypokalemic (2.7 mmol/L). She initially had a slight troponin leak that peaked at 0.05 ng/mL and a BNP of 206 pg/mL. An EKG showed no acute pathology. CT pulmonary angiography showed no evidence of pulmonary embolism but new extensive peribronchial vascular ground glass opacities and internal and interlobular septal thickening (Figure 1). Given her clinical symptoms, laboratory values, and imaging findings, she was admitted for treatment of community-acquired pneumonia. IV ceftriaxone and oral azithromycin were initiated, and an infectious workup was begun. This included a urinalysis with reflex urine culture, a urine antigen study, a sputum culture, a nasal methicillin-resistant *Staphylococcus aureus* (MRSA) test, and a viral respiratory panel that tested for influenza, parainfluenza, rhinovirus, metapneumovirus, respiratory syncytial virus, and adenovirus. Blood cultures were obtained when she became febrile on Hospital Day 2 (HD#2).

On HD#2, she continued to have increasing O_2_ requirements with desaturations to mid-80% SaO_2_ on pulse oximetry. Chest X-ray (CXR) revealed worsening airspace opacities. She was started on continuous positive airway pressure after a trial of a high-flow nasal cannula. Her symptoms did not improve. On HD#3, the intensive care unit (ICU) service was consulted, and she was transferred to the ICU for respiratory failure in the setting of likely noncardiogenic pulmonary edema (NCPE) and cryptogenic organizing pneumonia. A repeat CXR four hours later showed increased opacities in her right basilar lung. Her empiric antibiotic regimen was broadened to IV cefepime. Hydrocortisone 50 mg every 6 hours and IV furosemide were additionally begun.

In the ICU on HD#4, she transitioned to bilevel positive airway pressure ventilation. She remained tachypneic with respiratory rates over 30 breaths per minute and hypoxic receiving fraction of inspired oxygen (FiO_2_) at 90%. An arterial blood gas revealed a pH of 7.32, partial pressure of oxygen (PaO_2_) of 62 mmHg, partial pressure of carbon dioxide of 39 mmHg, and bicarbonate of 19.6 mmol/L. Her anion gap was normal, consistent with a primary metabolic acidosis with respiratory compensation. Additionally, given her arterial hypoxemia with a PaO_2_/FiO_2_ ratio of 68.89 mmHg, acutely worsening respiratory symptoms, bilateral pulmonary opacities on CXR and CT, and absence of left heart failure on echocardiogram, she met diagnostic criteria for acute respiratory distress syndrome (ARDS). Her hydrocortisone was increased to 100 mg every 6 hours, sulfamethoxazole/trimethoprim and azithromycin were initiated for coverage of atypical pneumonia, and diuresis was continued for NCPE/ARDS.

She was ultimately intubated on HD#5. Bronchoscopy with bronchoalveolar lavage (BAL) was performed to assist with elucidating an etiology. BAL cytology was notable for a neutrophilic predominance with serial bloody aliquots suggestive of diffuse alveolar hemorrhage (DAH). Further workup using BAL cytology included testing for fungus (*Histoplasma* species, *Aspergillus* species, *Candida* species, and fungal culture), bacteria (*Pneumocystis jirovecii*, *Pseudomonas aeruginosa*, MRSA, *Nocardia* species, *Legionella* species, *Mycobacterium* species, and bacterial culture), and vasculitides (autoimmune-mediated, immune complex-mediated, and antineutrophil cytoplasmic antibody-associated). These all returned negative. Given all of these results and input from the multiple clinical services involved in her care, her condition was attributed to grade 4 pneumonitis from G/D. She remained on her steroid regimen to treat her G/D-induced pneumonitis, and her empiric antibiotics were discontinued when the bacterial studies resulted.

**Figure 2:**
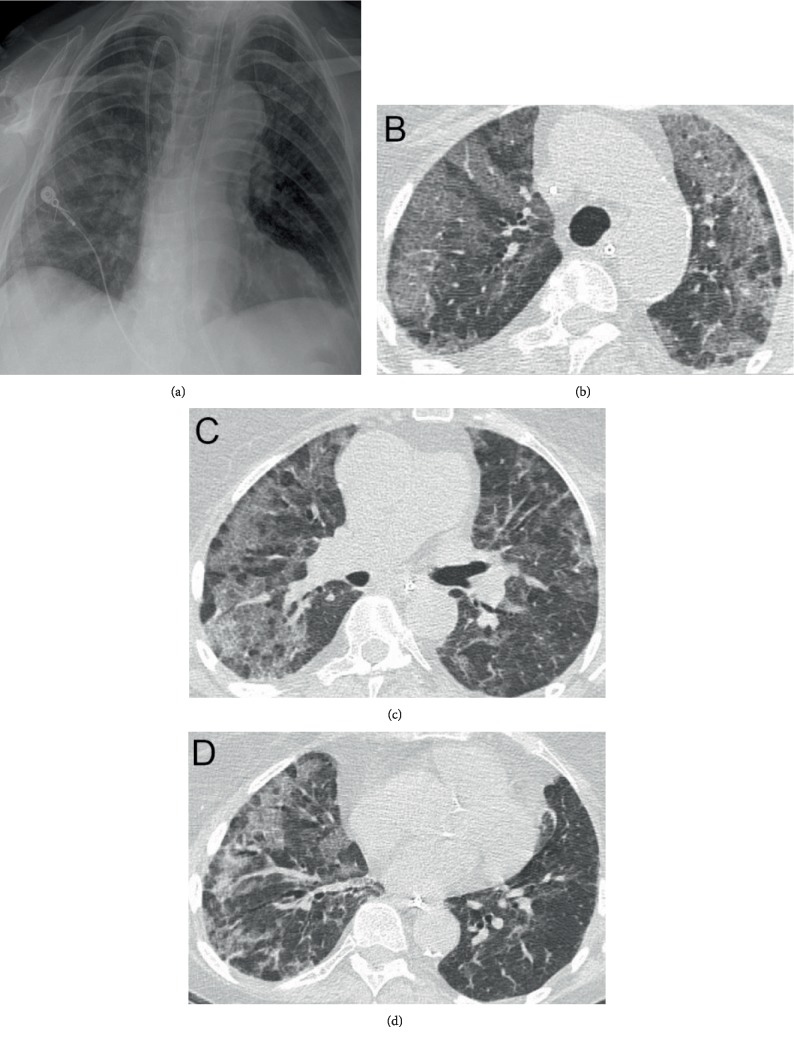
Imaging on HD#13. (a) CXR with continued bibasilar opacities and edema. No effusion. (b–d) CT scan of the lungs at upper (b), mid (c), and base (d) illustrating increased severity of ground glass airspace opacities.

On HD#7, she was extubated to a high-flow nasal cannula at 40 L/min of oxygen and slowly transitioned to a face mask on 3 L/min of oxygen over seven days. A repeat chest CT on HD#13, ten days after starting steroid therapy, revealed worsening ground glass airspace opacities (Figure 2). An extended steroid taper was initiated and diuresis continued. Her diet was advanced to clear liquids, which she tolerated initially.

**Figure 3:**
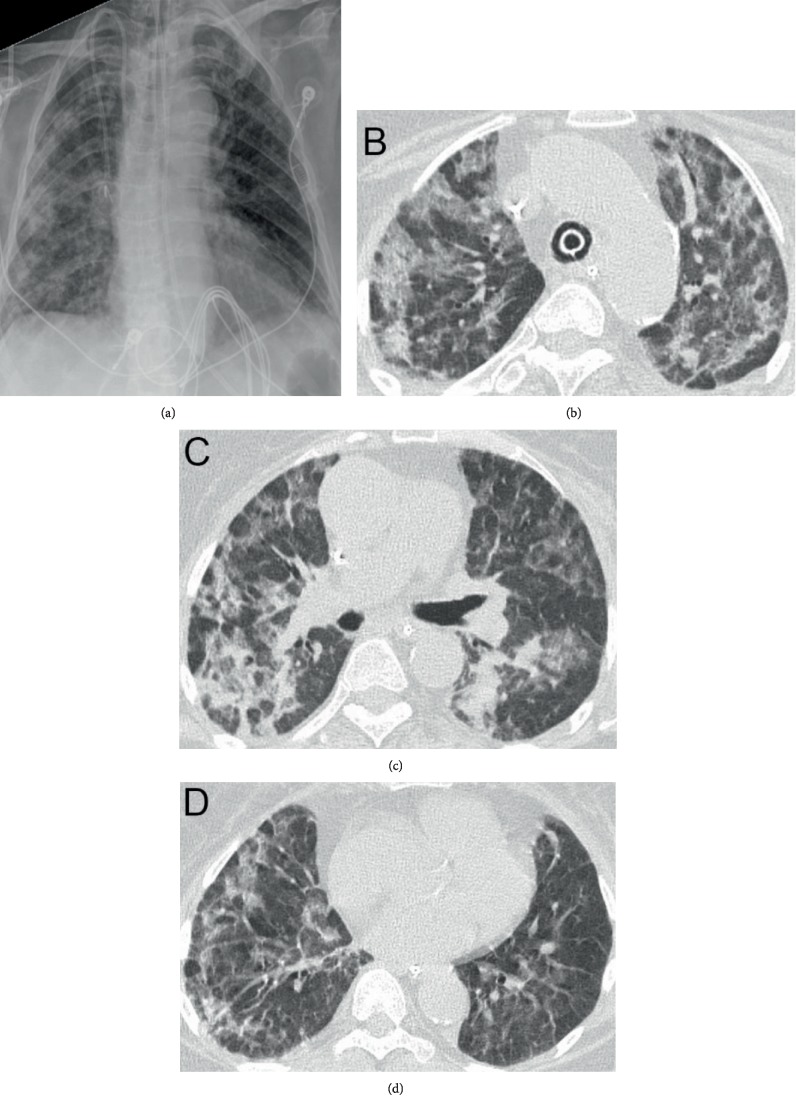
Imaging on HD#16 after an acute hypoxic event. (a) CXR with a new endotracheal tube and persistent diffuse lung disease. (b–d) CT scan of the lungs at upper (b), mid (c), and base (d) illustrating continued peribronchial consolidation and decreased ground glass opacities. An endotracheal tube can be seen in the trachea.

**Figure 4:**
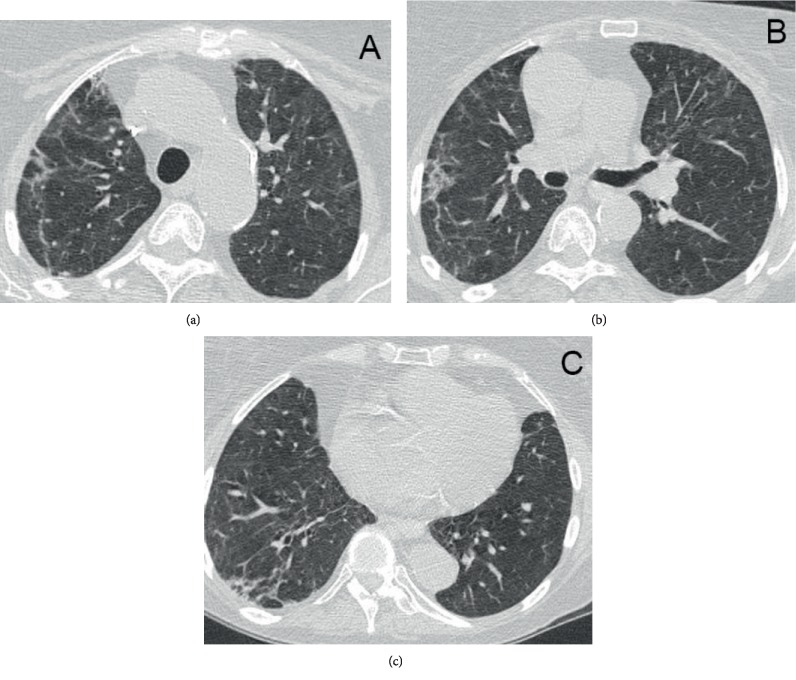
Imaging on 48 days after a discharge CT scan of the lungs at upper (a), mid (b), and base (c) illustrating improved residual ground glass and reticular opacities bilaterally.

On HD#16, she had an acute hypoxemic event requiring an emergent intubation and was transferred back to the ICU. Repeat chest CT on HD#17 showed worsening lung injury (Figure 3). She was extubated on HD#18 but required subsequent reintubation. Her steroid regimen was escalated to methylprednisolone 60 mg IV twice daily. On HD#22, given her worsening hypoxemic respiratory failure, multiple failed extubations, and difficulty weaning her from ventilatory support, a tracheostomy was performed. On HD#24, she was tolerating intermittent tracheostomy mask alternating with pressure-regulated volume control for rest periods. She was discharged to a long-term acute care hospital (LTACH), where she stayed for approximately 3 weeks and was weaned from mechanical ventilation and had her tracheostomy decannulated. At the time of discharge from the LTACH, she was saturating consistently at or above 95% on room air and tolerating a general diet. She was transferred to inpatient rehabilitation where she stayed for 2 weeks for comprehensive rehabilitation. Repeat chest CT 5 weeks after discharge demonstrated improved bilateral ground glass and reticular opacities. There were no findings of intrathoracic metastatic disease (Figure 4). At her 6-week posthospitalization follow-up, her Eastern Cooperative Oncology Group performance status was grade 2.

## 3. Discussion

Our case illustrates a rare occurrence of G/D-associated grade 4 pneumonitis presenting with rapidly progressing pulmonary failure in a patient with recurrent uLMS. In general, these pulmonary side effects can be variable in clinical presentation, severity, time from chemotherapy initiation, and clinical response to appropriate therapy for lung injury. They can manifest as mild to severe dyspnea, NCPE, DAH, ARDS, and interstitial pneumonitis (IP) [7–12]. Our patient, despite appropriate treatment with early steroid therapy, declined clinically and radiographically. Boiselle et al. [9] described the common CT imaging features in three patients with gemcitabine-induced lung injury. The predominant CT finding was ground glass attenuation accompanied by thickened septal lines and reticular opacities. None had suggestions of cardiogenic pulmonary edema [7, 9]. This pattern was consistent with the findings in our patient's CT scans (Figures 1–3) throughout her hospitalization.

The mechanism for gemcitabine-induced pulmonary toxicity is unknown, but it is thought to be due to drug-related increased capillary permeability leading to cytokine-mediated inflammatory responses [7, 8]. As a result, pulmonary toxicities associated with gemcitabine use can range from mild bronchospasms with dyspnea that self-resolve or resolve quickly with steroids to fatal injuries, including NCPE, interstitial pulmonary damage, alveolar wall inflammation and scarring, alveolar hemorrhage, and ARDS [7–10]. The mechanism of lung injury caused by gemcitabine is similar to the proposed method by which taxanes and docetaxel lead to pulmonary toxicity. Docetaxel is known to cause fluid accumulation in peripheral tissues, pleura, or peritoneum via capillary leakage, which induces a hypersensitivity reaction. This syndrome worsens with increased cycles of docetaxel, and premedication with steroids can decrease this response. Though docetaxel-induced pneumonitis has not been well described [11, 13], coadministration with gemcitabine may potentiate pulmonary inflammation and toxicity.

Risk factors for pulmonary toxicities have been hypothesized and include thoracic metastases, smoking history, prior or concurrent radiation therapy, and the use of multiagent therapy (including the G/D combination) [7]. In our patient, her only known risk factor was receipt of combination chemotherapy. Her pulmonary toxicity was likely due to G/D potentiating and augmenting cytokine release and the resultant lung damage.

Within the literature, there is a paucity of case reports on this chemotherapy combination and its associated adverse pulmonary events. One case report described a patient who developed likely grade 3 pulmonary toxicity while receiving G/D for metastatic primary ovarian LMS. She completely improved with steroid therapy and continued to receive the same chemotherapy regimen with a partial cancer response [12]. In a report of three patients with metastatic urothelial carcinoma treated with G/D, two had at least grade 3 pulmonary toxicity. One of those two patients died from respiratory failure with post mortem examination revealing changes consistent with DAH, and the other responded to high-dose steroids and recovered [13].

Although not detailed in case reports, studies investigating the use of G/D for uLMS have reported pulmonary toxicities as well. However, whether these instances were directly related to G/D is unknown. In a phase II trial of 34 patients with unresectable uLMS treated with G/D, 7 had at least grade 3 dyspnea [14]. In the arm of GOG-277 in which patients received gemcitabine plus docetaxel followed by doxorubicin for early-stage uLMS, grade 3 dyspnea was seen in 1 of the 17 patients [2]. GOG-87L [4], which trialed G/D in 39 women with advanced, unresectable uLMS without prior cytotoxic chemotherapy exposure, reported one grade 4 hypoxia and no additional grade 3 events. In that study, the patient had pulmonary metastases and previous thoracic radiation. Although she improved after treatment for possible *Pneumocystis jirovecii* pneumonia, she was removed from the study for possible G/D-related pulmonary toxicity. In GOG-131G [5], the investigators enrolled 48 metastatic uLMS patients who experienced cancer progression after one prior cytotoxic chemotherapy regimen. They had four grade 3 or worse pulmonary toxicities; however, none had clinical or radiographic evidence of G/D-induced pneumonitis, as their patients had attributable causes to their pulmonary toxicities, which included pneumonia with and without hypoxia, bilateral pleural effusion, and acute dyspnea and hypoxia during chemotherapy infusion. Similarly, in a study using G/D for advanced or recurrent uLMS and undifferentiated endometrial sarcoma in Japan, none of their 8 uLMS patients experienced pulmonary toxicity [15].

Severe lung toxicity due to chemotherapeutic agents is a diagnosis of exclusion. Workup should include tests and radiographs to exclude infectious, cardiogenic, vascular, oncologic, and drug-induced etiologies. In our patient and in several cases described in the literature, the initial treatment steps should include supportive therapy with nebulizers and supplemental oxygen, discontinuation of chemotherapeutic agents, and early steroid administration [8–11, 13]. The literature reports a range of clinical response to these interventions. Treatment response ranged from significant improvement with resolution of pulmonary toxicity to rapid progression and to respiratory failure and death [8–13]. Fenocchio et al. described a case of pancreatic adenocarcinoma treated with gemcitabine where the patient developed severe gemcitabine-induced pulmonary toxicity refractory to steroids and other conventional treatments. However, the patient responded completely to imatinib mesylate, a tyrosine-kinase inhibitor with antineoplastic activity. Imatinib mesylate has been studied as a potential treatment for lung fibrosis and may demonstrate a role in reversing gemcitabine-induced lung injury, but more studies are required before recommending widespread use [16]. Our patient was treated with high-dose steroid therapy early, starting on HD#3. This was increased once a diagnosis of chemotherapy-associated pneumonitis was made. Unfortunately, even though she received the appropriate interventions, her symptoms developed and progressed rapidly over her hospital course, which culminated in multiple intubations and ultimately a tracheostomy.

In the case of recurrent uLMS, the standard of care is G/D combination therapy. However, they are both widely used and generally well-tolerated chemotherapy agents [2–6]; prescribers should be cognizant of this rare, life-threatening pulmonary toxicity that we describe for the first time in a patient with recurrent, metastatic uLMS.
